# Radial Endobronchial Ultrasound for Lung Cancer Diagnosis: Tips and Tricks

**DOI:** 10.7150/jca.67113

**Published:** 2022-01-31

**Authors:** Paul Zarogoulidis, Haidong Huang, Wei Chen, Dimitris Petridis, Dimitris Matthaios, Wolfgang Hohenforst-Schmidt, Christos Tolis, Kosmas Tsakiridis, Sofia Baka, Christos Arnaoutoglou, Maria Saroglou, Stavros Tryfon, Aris Ioannidis, Lutz Freitag, Christoforos Kosmidis, Chong Bai

**Affiliations:** 1 3rd Department of Surgery, “AHEPA” University Hospital, Aristotle University of Thessaloniki, Medical School, Thessaloniki, Greece.; 2 Pulmonary Oncology Department, “Bioclinic” Private Hospital, Thessaloniki, Greece.; 3 Department of Respiratory & Critical Care Medicine, Changhai Hospital, the Second Military Medical University, Shanghai, China.; 4 Department of Respiratory and Critical Care Medicine, The Huaian Clinical College of Xuzhou Medical University, Huai'an, Jiangsu, China.; 5 Department of Food Technology, School of Food Technology and Nutrition, Alexander Technological Educational Institute, Thessaloniki, Greece.; 6 Oncology Department, General Hospital of Rhodes, Rhodes, Greece.; 7 Sana Clinic Group Franken, Department of Cardiology/Pulmonology/Intensive Care/Nephrology, ''Hof'' Clinics, University of Erlangen, Hof, Germany.; 8 Oncology Department, “Oncoderm” Private Oncology Clinic, Ioannina, Greece.; 9 Thoracic Oncology Department, “Interbalkan” European Medical Center, Thessaloniki, Greece.; 10 Oncology Department, “Interbalkan” European Medical Center, Thessaloniki, Greece.; 11 1st Department of Obstetrics & Gynecology, Papageorgiou Hospital, Aristotle University of Thessaloniki, Periferiakos Str., 56429, Thessaloniki, Greece.; 12 Pulmonary Department, “G. Papanikolaou” General Hospital, Aristotle University of Thessaloniki, Thessaloniki, Greece.; 13 Surgery Department, Genesis Private Hospital, Thessaloniki, Greece.; 14 Department of Pulmonology, University Hospital Zurich, Zurich, Switzerland.

**Keywords:** radial ebus, lung cancer, 22G needle, forceps, brush, C-Arm, nsclc, sclc, metastasis, elastography

## Abstract

**Introduction:** Endoscopic techniques have been upgraded in the recent 10 years. We can use the radial endobronchial ultrasound to reach distal nodules in the periphery of the lungs, but also we can use it in order to make biopsies in lesions without endobronchial findings.

**Patients and Methods:** We included in our study 248 patients with pulmonary nodules up to 4 cm. We use a radial endobronchial system from FUJI, a PENTAX bronchoscope and a C-ARM. We recorded the cancer type, biopsy method, time of each procedure, cell blocks and slices from cell blocks.

**Results:** Two thirds of patients belonged to males (61.7%), forceps was the main tissue extraction technique (118, 47.6%) and tumors sized 1 to 2 cm were the most encountered (96, 38.7%). Samples with tissue content were present in 175 patients (70.6%) and one cell block dominated in the samples (109, 43.9%). Less than 20 minutes were needed to complete the operative procedure for the half patients (127, 51.2%), the C-Arm implementation concerned 117 persons (47.2%) and the majority of tumors was located in the central area of the lungs (178, 71.8%). Less time was necessary for central lesions and larger biopsy samples were acquired without the extensive use of C-ARM.

**Conclusion:** The larger the nodule ≥2cm and in periphery the less we use the C-ARM and the time of the procedure is between 20-40 minutes. Moreover; we have more tissue sample and cell block slices.

## Introduction

Lung cancer is still diagnosed at a late stage due to lack of early disease symptoms. In the past ten years an effort has been made towards lung cancer screening 1. The screening program is focused on patients of ≥50 years of age with an active smoking habit or previous tobacco use. Additional, groups of patients are included in the screening program such as occupational exposure to toxic particles. The screening is performed with low-dose computed tomography (CT) without the addition on contrast intravenously, although this can be done if there are suspicious findings. Therefore more and more patients are diagnosed in the everyday practice with early disease in the form usually of nodules. Possitron emission tomography (PET-CT) usually follows in order to have information regarding the activity of the nodule or nodules. The next and most important step for diagnosis is the biopsy of such pulmonary findings 2, 3. The guidelines are clear, in the case of single pulmonary nodule ≤3cm in the periphery of the lungs, positive PET-CT SUV≥3 and negative results from the mediastinum lymphnodes, surgery is proposed. For any other finding or situation then staging is required with convex-probe endobronchial ultrasound for proper lung cancer staging, PET-CT findings alone are not enough 3. In order to perform a biopsy to peripheral lesions/nodules we can use the radial endobronchial ultrasound 4-6. We can also add the use of a C-Arm in order find a small nodule in the periphery faster and easier. Through the past 10 years, additional navigation systems have been included in the market such as the ARCHEMEDES®, superDimension™ 7, 8. Based on the location of the lesion several tools can be used for biopsy, such as; forceps, needle and brush. Cone-Beam CT is another tool for real time navigation and larger biopsy needles such as; 18G and 19G can be used. Also, in the case of pneumothorax, a pleurocath can be inserted on site. Based on a recent review and meta-analysis of 41 studies comparing radial-ebus and electromagnetic navigation it was concluded that both technologies have a high proportion of successful PPL localization with similar sensitivity for malignancy and accuracy. As such, both reasonable options for health care authorities to employ diagnostic algorithms 9. We will present our experience regarding the nodule size and biopsy technique with the navigation systems of radial ultrasound and C-ARM in order to provide useful information regarding the best combination regarding time of the procedure and effectiveness.

## Patients and Methods

### Patients

We included 248 patients with pulmonary nodules. Our patients were admitted for diagnosis of pulmonary nodules, (primary lung cancer or metastatic cancer). Patients inclusion were nodules up to 4cm and were undiagnosed. All patients were fit to have sedation and have the endoscopic diagnostic procedure. We had performed in all patients only computed tomography of the thorax without i.v contrast. PET-CT was performed after the biopsy.

## Methods

**Figure 1:**
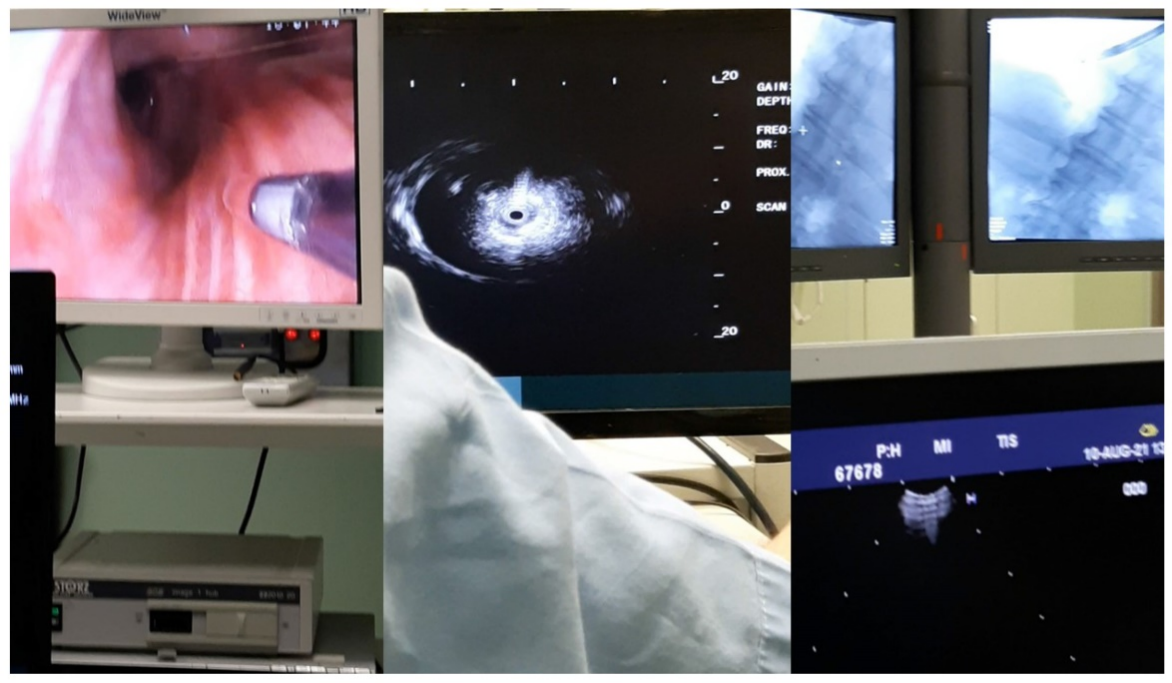
Left; endoscopic bronchoscopic image with the radial-ebus outside the bronchoscope, middle; image of the radial-ebus presenting a nodule, right; C-ARM image presenting the endoscope within the right bronchus.

**Figure 2:**
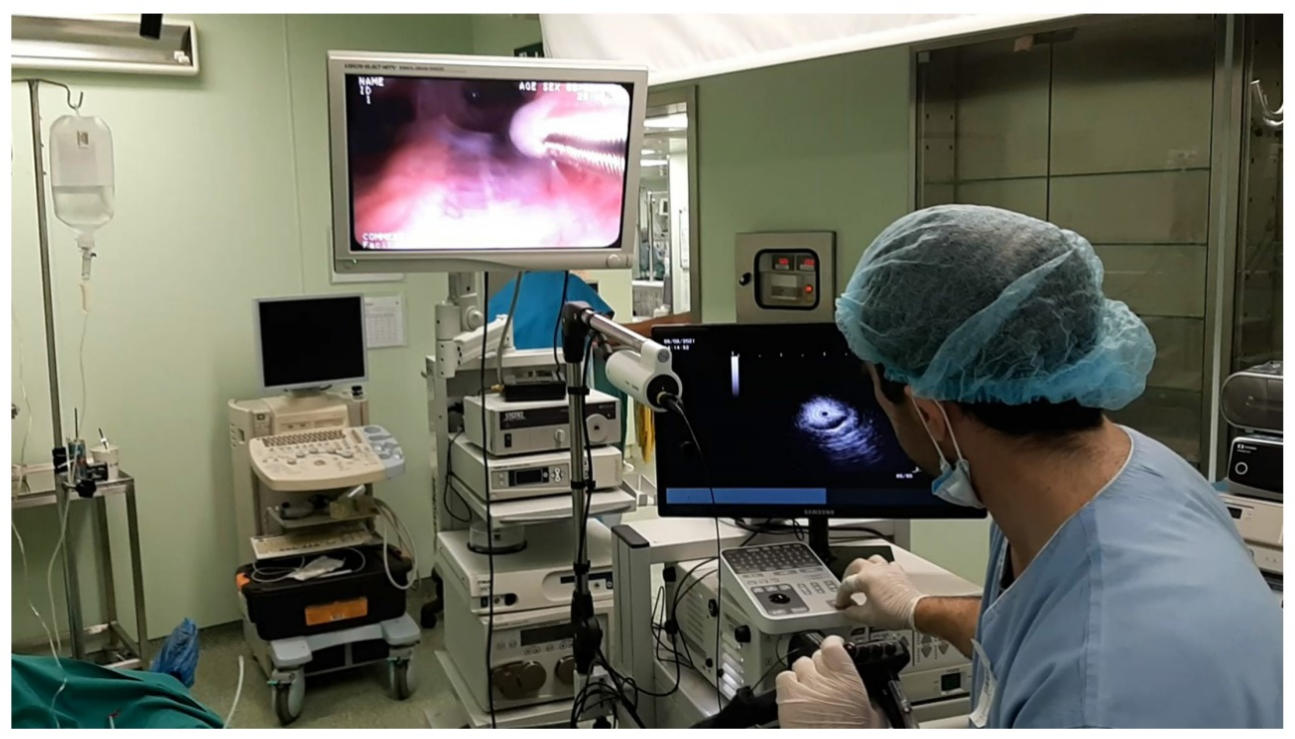
During a procedure.

Inclusion criteria were all patients ≥18 years of age with nodules and suspicion of cancer. Moreover; all patients were fit to have endoscopic procedures. All patients CT of the thorax without i.v. contrast prior to the endoscopic examination. We used a PENTAX bronchoscope with a 2.8 mm working channel. A radial endobronchial ultrasound from FUJI and a SIEMENS C-ARM. Moreover, we recorded the time of each procedure, number of cell blocks produced from each patients and how many patients had only or higher tissue sample. Furthermore; we recorded the cancer type, location of the nodules (central or peripheral), biopsy methodology, nodule size (cm). We recorded also the age and sex of the patients. All patients had sedation and were under jet-ventilation during the diagnostic procedure. Figures 1-2.

### Statistical analysis methodology

Tumor size and location in the lung tissue were considered the key-variables under study. The former was regressed as an ordinal response against a set of candidate independent variables: method of tissue detachment, number of cell blocks and slices, presence of tissue in the sample, total duration of operation and use of C-Arm. The latter was checked for possible association with operative time and use of C-Arm via tests of independence (Pearson's and likelihood ratio chi-square, Fisher's exact test). In all statistical tests the probability level of 0.05 was taken as the reference value.

## Results

**Figure 3:**
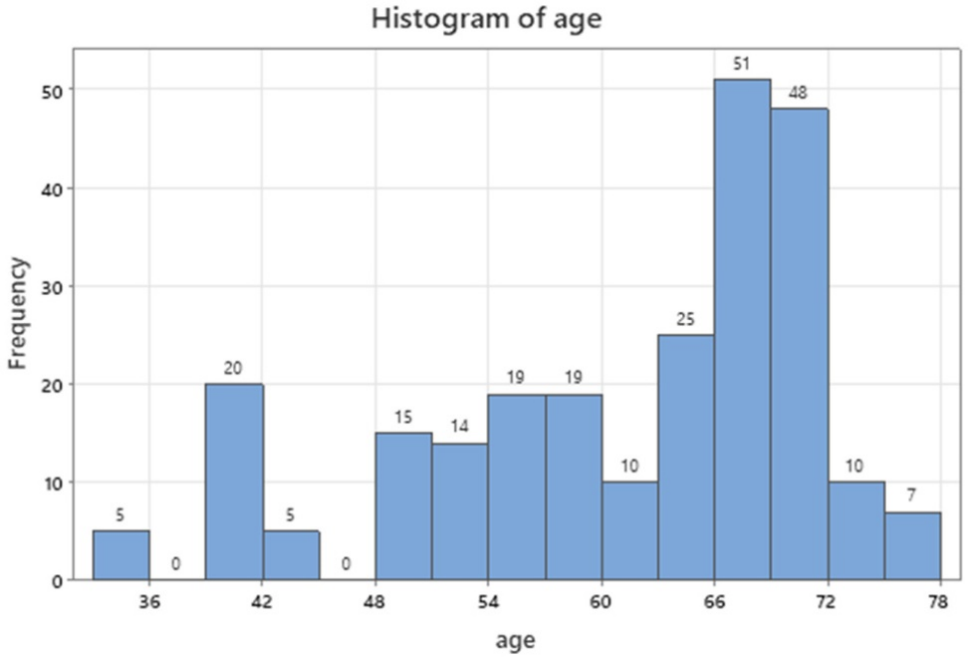
Age distribution of patients.

Age of patients ranged between 33 and 78 y.o. (**Table 1**) producing a prevailing mode of sixties to seventies in the age distribution of Figure 3 (96, 38.7%).

**Figure 4:**
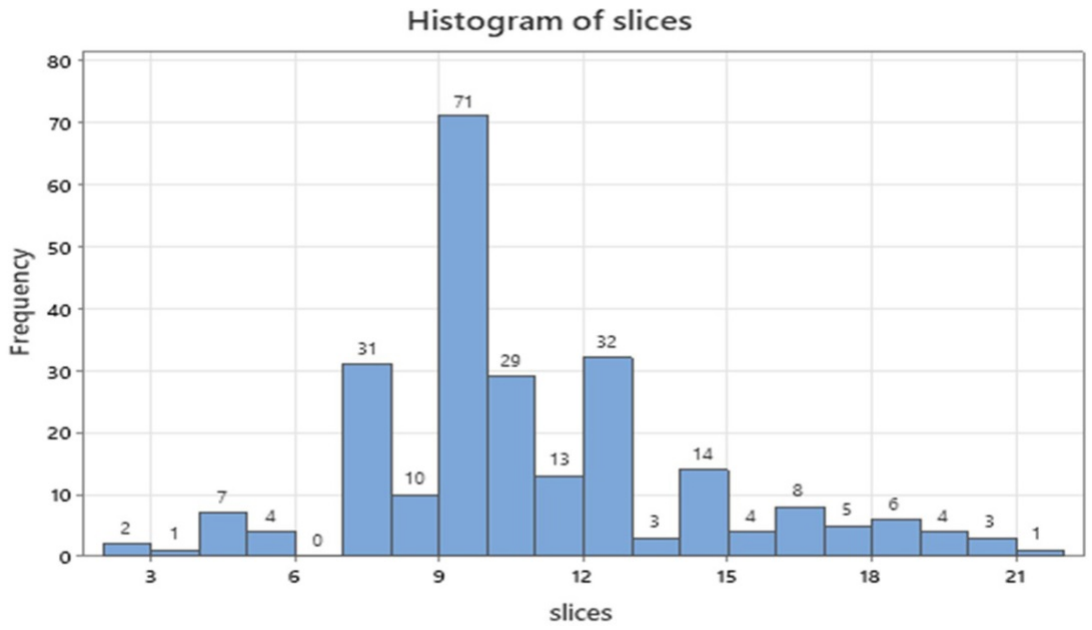
Numerical distribution of slices under study.

In **Supplementary Table 2**, two thirds of patients belonged to males (61.7%), forceps was the main tissue extraction technique (118, 47.6%) and tumors sized 1 to 2 cm were mostly encountered (96, 38.7%). Samples with tissue content were present in 175 patients (70.6%) and one cell block dominated in the samples (109, 43.9%). Less than 20 minutes were needed to complete the operative procedure for the half patients (127, 51.2%), the C-Arm implementation concerned 117 patients (47.2%) and the majority of tumors was located in the central area of the lungs (178, 71.8%). Ten slices were most often dissected for diagnosis (71 cases, 28.6%, Figure 4) accompanied by 8, 11 and 13 slices totaled 37.5% (92 patients).

The tumor location was significantly connected with the C-Arm usage (Supplementary Figure 5) as the tests of independence showed too high chi-square values and low exact probability values (p<0.0001).

These results were further clarified by the Fisher's exact test, according which the peripheral tumors were more frequently radiated (54 cases as compared to 16 ones). On the contrary, the C-Arm performance was not needed in 115 cases with central tumors while only 63 patients were X-rayed. In terms of relative risks concerning the previous findings, it appears that patients with central lung tumors have 1.6 times higher probability to avoid the C-Arm performance (P(1|0)/P(1|1) and those with outer lung tumors have 3.8 times higher probability to be radiated by C-Arm (P2|1/P2|0). Globally, patients spotted with the above conditions have jointly 6.2 times higher probability to happen as compared to those with inverse conditions.

The operative time was significantly affected by the tumor location (Supplementary Figure 6) as the tests of independence so indicated.

The individual chi-square statistics are greater than 3.841 for the number of cell slices between 12 and 22, meaning that at time interval of 20 minutes lower observed counts with peripheral location were recorder than expected (-19.8 difference) and at time interval of 21-40 minutes higher observed counts were found than expected (13.2 difference). Recalling the Cochran Armitage test for detection of trends, it appears that a decrease of counts for operative time develops for patients only with centrally spotted tumors (prob Z<0.0001). In other words, most of the outer lung tumors need 21-40 minutes to be fully operated/diagnosed. This test is appropriate only when one nominal and one ordinal variable are considered.

There is an imperative questioning on how to best predictably accomplish an operation on lung tumors whose size varies between minimum and 4 cm. An attempt was conducted by regressing the four sizes (see **Table 1** against a set of candidate variables to enter the model by performing a stepwise forward selection of variables (p-enter≤0.05). The stepwise results are shown in **Supplementary Table 2**, in which “slices” was discarded, C-Arm was the most important (first variable to enter in the model), stressing also that cell blocks (0-2) and method (4-3) little contributed to the final McFadden R^2^ (3.74%).

The ordinal regression assumes a proportional log odds pattern between the ranks of the response variable and inspecting at the odds ratios in the parameter estimates the following eventuate: Sizes represent the intercepts of the response (size 4 not shown) and the odds ratios are calculated as antilog of the regression coefficients.

When the cell blocks 0 and 2 are grouped together, they have 51.42 times higher probability of occurrence as compared to the group of cell blocks 1 and 3. This is in accordance with the fact that larger nodules were easier to be approached and larger tissue samples were acquired. Also, the effect of cell block 0 as compared to present 2 blocks is expected to occur 14.0 times more often than the latter. This is in accordance with the facts that in those cases were nodules were in the periphery and ≤2cm the operator used 3 biopsy techniques in order to be sure of the biopsy result.

The operative procedure with tissue content present in the samples, has 66.7 times higher probability to occur than with empty content (66.7=1/0.015).

The operative time interval of 21-60 minutes is encountered 11.2 times more frequently than the short interval (-20 minutes). Additionally, the high time interval (41-60 minutes) is expected to happen 3.8 more times more than the middle interval (21-40). The C-Arm performance is executed 3.0 times more often than the times of no use (3.03=1/0.33). The combo method of detachment is implemented 3.5 more times than that of brush use. The reliability of the regression model is depicted by the misclassification rate of the confusion matrix between the actual and predicted values, which reaches only 7.2%. Size 1 fails to be predicted in 10 out of 68 cases, size 2 in 3 out of 96, size 3 in 4 out of 66 and size 4 fails only in 1 case out of 18 observations.

The ordinal regression model despite its high reliability due to the low misclassification rate and the high determined R^2^ should be checked for its validation via repetitive real time operative procedures.

## Discussion

Pulmonary nodules have been and are a diagnostic issue for several years. Usually they are benign however; in several cases they are malignancies with low ki-67. We have to take a careful medical history. Smoking habit is certainly an issue and even small nodules ≤2cm should be considered suspicious. In the case where patients had previously been diagnosed with malignancy other than lung cancer then metastasis should be considered. Rapid on site evaluation (ROSE) should be considered in the case of possible primary lung cancer, otherwise we need immunohistochemistry to have optimal diagnosis 10. Radial endobronchial ultrasound and other navigation systems can provide rapid evaluation for pulmonary nodules and therefore since this technology is available more diagnostic procedures should be performed instead of performing computed tomography scans or PET-CTs every 2-3 months. PET-CTs can provide information regarding the nature of the lesion, in the case of malignancy high SUV≥3 will be recorded. However; PET-CT has its limitations which mainly have to do with the size and ki-67 percentage. We concluded that the larger the lesion ≥2 cm and central located the less the time of the procedure ≤40 minutes and less the usage of C-ARM. Moreover; the larger the lesion ≥2 cm the more tissue is observed in the sample and more slices are created from the paraphin blocks. In the case of small nodules ≤2 cm all biopsy tools should be used with forceps being the best and 22G needle should be carefully used because of the adverse effects that they might induce. It should be mentioned that based on a recently published study 11 22G needle have very few adverse effects. However; in several situations we will have to puncture through normal lung tissue in order to acquire sample from the lesion.

## Supplementary Material

Supplementary figures and tables.

## Figures and Tables

**Table 1:** A tally of the categorical parameters and descriptive statistics of the quantitative variables. Method: Number 1 represents the needle biopsy tool, number 2 represents the forceps biopsy tool, number 3 represents the brush biopsy tool and number 4 represents the combination of forceps and brush biopsy tool. Tissue: 0 represents the absence of tissue while 1 tissue fragments. Time: 1 indicates that the length of the procedure was between 0-20minutes, 2 indicates that the length of the procedure was between 21-40 minutes and 3 indicates that the length of the procedure was between 41-60 minutes. C-Arm: 0 indicates that no C-Arm was used, while 1 indicates that C-Arm was used. Location: 1 indicates that the lesion was central, while 1 indicates that the lesion was in the periphery of the lung.

Frequencies sex	Count	Percent	Method	Count	Percent	Size (cm)	Count	Percent
0 (female)	95	38,31	1 (needle 22G)	49	19,76	1 (0-1)	68	27,42
1 (male)	153	61,69	2 (forceps)	118	47,58	2 (1-2)	96	38,71
N=	248		3 (brush)	13	5,24	3 (2-3)	66	26,61
			4 (combo)	68	27,42	4 (3-4)	18	7,26
			N=	248		N=	248	
**Tcell blocks**	**Count**	**Percent**	**Tissue**	**Count**	**Percent**	**Time**	**Count**	**Percent**
0	68	27,42	0 (No)	73	29,44	1 (20)	127	51,21
1	109	43,95	1 (Yes)	175	70,56	2 (21-40)	70	28,23
2	50	20,16	N=	248		3 (41-60)	51	20,56
3	21	8,47				N=	248	
N=	248							
**C-Arm**	**Count**	**Percent**	**Location**	**Count**	**Percent**			
0 (No)	131	52,82	1 (central)	178	71,77			
1 (Yes)	117	47,18	2 (peripheral)	70	28,23			
N=	248		N=	248				

Descriptive Statistics.
