# Supplementary material for: Radial Endobronchial Ultrasound for Lung Cancer Diagnosis: Tips and Tricks
Source: J Cancer. 2022 Jan 31;13(4):1307–12. doi: 10.7150/jca.67113 (PMC8899369; doi:10.7150/jca.67113)
Supplement: Supplementary file 1 — Supplementary figures and tables. [file jcav13p1307s1.pdf]

Supplementary material

A. Contingency analysis. Each cell contains the observed and expected counts, their difference and the chi-square statistic whose value greater than 3,841 show significant result.

Contingency Table

C-Arm By location

Count<br>Expected<br>Deviation<br>Cell Chi^2	1	2	Total
0	115<br>94,0242<br>20,9758<br>4,6795	16<br>36,9758<br>-20,976<br>11,8993	131
1	63<br>83,9758<br>-20,976<br>5,2394	54<br>33,0242<br>20,9758<br>13,3231	117
Total	178	70	248

B. Tests of independence

Test	ChiSquare	Prob>ChiSq
Likelihood Ratio	36,408	<,0001
Pearson	35,141	<,0001

Fisher's Exact Test	Prob	Alternative Hypothesis
Right	<,0001	Prob(location=2) is greater for C-Arm=1 than 0
2-Tail	<,0001	Prob(location=2) is different across C-Arm

C. Relative Risk

Description	Relative Risk	Lower 95%	Upper 95%
P(1|0)/P(1|1)	1,630316	1,362427	1,950879
P(1|1)/P(1|0)	0,613378	0,512589	0,733984
P(2|0)/P(2|1)	0,264631	0,160656	0,435898
P(2|1)/P(2|0)	3,778846	2,294116	6,224481

D. Odds Ratio

Odds Ratio	Lower 95%	Upper 95%
6,160714	3,258668	11,64721

Figure 5. Statistical report of cross-tabulated location by C-Arm.

A. Contingency analysis. Each cell contains the observed and expected counts, their difference and the chi-square statistic whose value greater than 3,841 show significant result.

Contingency Table

Time By location

Count<br>Expected<br>Deviation<br>Cell Chi^2	1	2	Total
1	111<br>91,1532<br>19,8468<br>4,3212	16<br>35,8468<br>-19,847<br>10,9883	127
2	37<br>50,2419<br>-13,242<br>3,4901	33<br>19,7581<br>13,2419<br>8,8748	70
3	30<br>36,6048<br>-6,6048<br>1,1918	21<br>14,3952<br>6,60484<br>3,0305	51
Total	178	70	248

B. Tests of independence

Test	ChiSquare	Prob>ChiSq
Likelihood Ratio	33,055	<,0001
Pearson	31,897	<,0001

C. Cochran Armitage Trend Test

Asymptotic Test

Z	Prob<Z	Prob>|Z|
-4,72483	<,0001	<,0001

Figure 6. Statistical report of cross-tabulated location by time.

Table 2. Step history of the forward selection of independent variables on tumor size: method, cell blocks, slices, tissue, time and C-Arm.

					size			
method	cell blocks	tissue	Time	C-Arm	(Prob[1])	(Prob[2])	(Prob[3])	(Prob[4])
1	0	0	2	1	0,3345	0,6602	0,0054	0,0000
1	1	0	1	0	0,0000	0,0000	0,0150	0,9849
1	1	1	1	0	0,0002	0,0653	0,9192	0,0154
1	1	1	2	1	0,0536	0,9007	0,0456	0,0001
1	2	0	1	0	0,0000	0,0031	0,7390	0,2579
1	2	1	1	0	0,0345	0,8950	0,0704	0,0001

1	2	1	1	1	0,2429	0,7487	0,0084	0,0000
1	2	1	2	1	0,9143	0,0855	0,0003	0,0000
1	3	1	1	0	0,0002	0,0653	0,9192	0,0154
2	0	0	1	0	0,0017	0,3816	0,6150	0,0018
2	0	0	2	0	0,0531	0,9008	0,0461	0,0001
2	0	0	2	1	0,3345	0,6602	0,0054	0,0000
2	1	1	1	0	0,0002	0,0653	0,9192	0,0154
2	1	1	2	0	0,0063	0,6933	0,2999	0,0005
2	1	1	2	1	0,0536	0,9007	0,0456	0,0001
2	1	1	3	1	0,4449	0,5517	0,0034	0,0000
2	2	1	1	0	0,0345	0,8950	0,0704	0,0001
2	2	1	1	1	0,2429	0,7487	0,0084	0,0000
2	2	1	2	1	0,9143	0,0855	0,0003	0,0000
2	2	1	3	1	0,9934	0,0066	0,0000	0,0000
2	3	1	1	0	0,0002	0,0653	0,9192	0,0154
3	1	1	2	1	0,0160	0,8413	0,1424	0,0002
3	1	1	3	1	0,1874	0,8010	0,0116	0,0000
3	2	1	2	1	0,7543	0,2448	0,0009	0,0000
4	0	0	2	1	0,6359	0,3626	0,0015	0,0000
4	0	0	3	1	0,9611	0,0388	0,0001	0,0000
4	0	1	2	1	0,9999	0,0001	0,0000	0,0000
4	0	1	3	1	1,0000	0,0000	0,0000	0,0000
4	1	0	3	1	0,0007	0,1961	0,7988	0,0044
4	1	1	2	1	0,1645	0,8219	0,0136	0,0000
4	1	1	3	1	0,7358	0,2632	0,0010	0,0000

Effect Summary

Source	LogWorth	PValue
cell blocks(0&2-1&3)	42,870	0,00000
tissue	19,269	0,00000
Time(3&2-1)	11,798	0,00000
Time(3-2)	4,087	0,00008
cell blocks(0-2)	3,023	0,00095
C-Arm	2,913	0,00122
method(4-3)	2,413	0,00387

Whole Model Test

Model	-LogLikelihood	DF	ChiSquare	Prob>ChiSq
Difference	228,18184	7	456,3637	<,0001
Full	85,50063			
Reduced	313,68247			

Fit Details

RSquare (U)	0,7274
AICc	191,93
BIC	226,13
Misclassification Rate	0,072

Parameter Estimates

Term	Estimate	Std Error	ChiSquare	Prob>ChiSq	Lower 95%	Upper 95%	Odds Ratio
Size[1]	-5,287	0,593	79,43	<,0001	.	.	
Size[2]	0,623	0,512	1,48	0,2237	.	.	
Size[3]	7,441	0,963	59,76	<,0001	.	.	
cell blocks(0&2-1&3)	3,940	0,475	68,91	<,0001	3,033	5,159	51,42
cell blocks(0-2)	2,643	0,798	10,97	0,0009	0,970	4,764	14,05
tissue[0]	-4,170	0,760	30,07	<,0001	-6,264	-2,705	0,015
Time(3&2-1)	2,414	0,372	42,09	<,0001	1,707	3,192	11,18
Time(3-2)	1,325	0,349	14,39	0,0001	0,656	2,060	3,76
C-Arm[0]	-1,097	0,369	8,83	0,0030	-1,906	-0,417	0,33
method(4-3)	1,246	0,437	8,11	0,0044	0,398	2,127	3,48

Confusion Matrix

Actual	Predicted Count			
size (cm)	1	2	3	4
1	58	6	4	0
2	1	93	2	0
3	0	4	62	0
4	0	0	1	17

Figure 7. Statistical output of the ordinal logistic regression of tumor size against the variables entered in the model by the forward selection (LogWorth=-logp value).

Table 3. Mean probability values of tumor size according to the combined effects of the independent variables.

Some individual patterns with probability values of occurrence higher that 0.800 are distinct from the table:

Size 1 occurs in high proportions in the variable combination:

cell blocks	tissue	Time	C-Arm
2	1	2(3)	1

and also in the pattern:

method	cell blocks	C-Arm
4	0	1

Size 2 is associated with:

cell blocks	tissue	Time	C-Arm
2	1	1	0(1)

and also

cell blocks	tissue	Time	C-Arm
1	1	2(3)	1

Size 3 is found in the coded values:

cell blocks	tissue	Time	C-Arm

3(1)	1	1	0

Size 4 is practically indicative in a single combined format:

method	cell blocks	tissue	Time	C-Arm
1	1	0	1	0

Numbers in brackets indicate an alternative occurrence.
